# Comparative Analysis of Child Sexual Abuse Survivors During the Lockdown and Pre-lockdown Periods of COVID-19 at a Tertiary Care Centre in North East India

**DOI:** 10.7759/cureus.92104

**Published:** 2025-09-11

**Authors:** Oli Goswami, Kaustav Bairagi, Arpan Mazumdar, Bhaskar Mukherjee, Bhaswat Das

**Affiliations:** 1 Department of Forensic Medicine and Toxicology, Employees' State Insurance Corporation (ESIC) Medical College and Hospital, Beltola, Guwahati, IND; 2 Department of Forensic Medicine and Toxicology, All India Institute of Medical Sciences, Guwahati, IND; 3 Department of Forensic Medicine and Toxicology, Nagaon Medical College and Hospital, Nagaon, IND; 4 Biostatistics, Regional Resource Centre for North East States, Ministry of Health and Family Welfare (MoHFW), Guwahati, IND

**Keywords:** child sexual abuse, covid 19, lockdown, pocso act, pre-lockdown

## Abstract

Background: Child sexual abuse is an issue of global concern, adversely affecting a child’s normal maturation and development. The COVID-19 pandemic caused a health and economic crisis, confinement of the masses, and sexual exploitation.

Purpose: This was a comparative analysis of the survivors under the POCSO Act (Protection of Children from Sexual Offences Act, 2012, Amdt.2019) between the lockdown and pre-lockdown periods of the same duration, in terms of number of cases, demographic details, reporting time, and patterns of injuries.

Methods: Hospital-based descriptive epidemiological study using secondary data of child sexual abuse survivors from 25/03/2017 to 14/02/2019 and from 25/03/2020 to 14/02/2022. Data distribution was represented in terms of frequencies and percentages. Association and effect size between the various input variables were analyzed using Cliff's delta method and chi-square test, considering a p-value of less than 0.05 to be statistically significant.

Results: There was a 53.7% increase in cases in the lockdown period. Consent rate for medical examination dropped from 70.9% to 63.1% during the lockdown. 96.7% and 97.9% were females in the lockdown and pre-lockdown periods, respectively. The highest number of cases was reported in 24-48 hours in the pre-lockdown period as compared to 48-72 hours in the lockdown period. The pre-lockdown period showed rural preponderance. There was a rise in survivors in the age group of zero to six years to 12.3% in the lockdown period. Known faces dominated as perpetrators in both periods.

Conclusion: The study revealed an alarming rise in child sexual abuse during COVID-19, calling for strict interventions. It is the duty of society to protect children's basic rights for a better and prosperous future.

## Introduction

Child sexual abuse has been a global challenge and a serious threat to humanity. The lockdown due to COVID-19 was introduced worldwide to limit the transmission of the disease. It was a period of social and economic instability, which also witnessed loosening of the social support system. Children were suddenly exposed to a life of prolonged school closure, switching to virtual media, social distancing and limited access to community support services. This exposed them to many hardships, including child sexual abuse. Abused children failed to seek help or escape from the situation. The resultant psychological effects were immense and unforeseen. Child Rights and You conducted a study on the report from the National Crime Records Bureau of India and found that during the pandemic, crimes against children increased by 16.2% [1]. In India, the Child Helpline recorded a 50% increase in call volumes during the last two weeks of the lockdown [2]. Out of the total calls, 30% were related to abuse [2].

## Materials and methods

This hospital-based descriptive epidemiological study was undertaken after getting approval from the Institutional Ethics Committee vide reference number Ref. No. MC/190/2007/Pt-II/Dec-2021/28, dated 10.01.2022, using secondary data of the survivors of child sexual abuse. Data was retrieved from preexisting records of the Department of Forensic Medicine of a tertiary care center, maintaining the confidentiality of the survivors’ identities throughout the study. Out of the totals of 538 and 350 child sexual abuse survivors registered during the lockdown and pre-lockdown periods, 340 and 248 consented to medico-legal examination, respectively. The duration of study for the lockdown period was taken from 25/03/2020 to 14/02/2022 and the corresponding pre-lockdown period from 25/03/2017 to 14/02/2019. The aims and objectives of the study were to do a comparative analysis of the child sexual abuse survivors under the POCSO Act (Protection of Children from Sexual Offences Act, 2012, Amdt. 2019) between the pre-lockdown and lockdown periods of the same duration in terms of the number of cases, demographic details, reporting time, and pattern of injuries.

Statistical analysis

The data were entered into Microsoft Excel (Microsoft Corporation, Redmond, WA, USA) and analyzed using the Statistical Package for the Social Sciences (SPSS) version 21 (IBM Corp., Armonk, NY, USA). The data distribution was represented in terms of frequencies and percentages. The association as well as the effect size between the various input variables of the lockdown and pre-lockdown period was analyzed using Cliff's delta method and the chi-square test, considering a p-value of less than 0.05 to be statistically significant.

## Results

**Figure 1:**
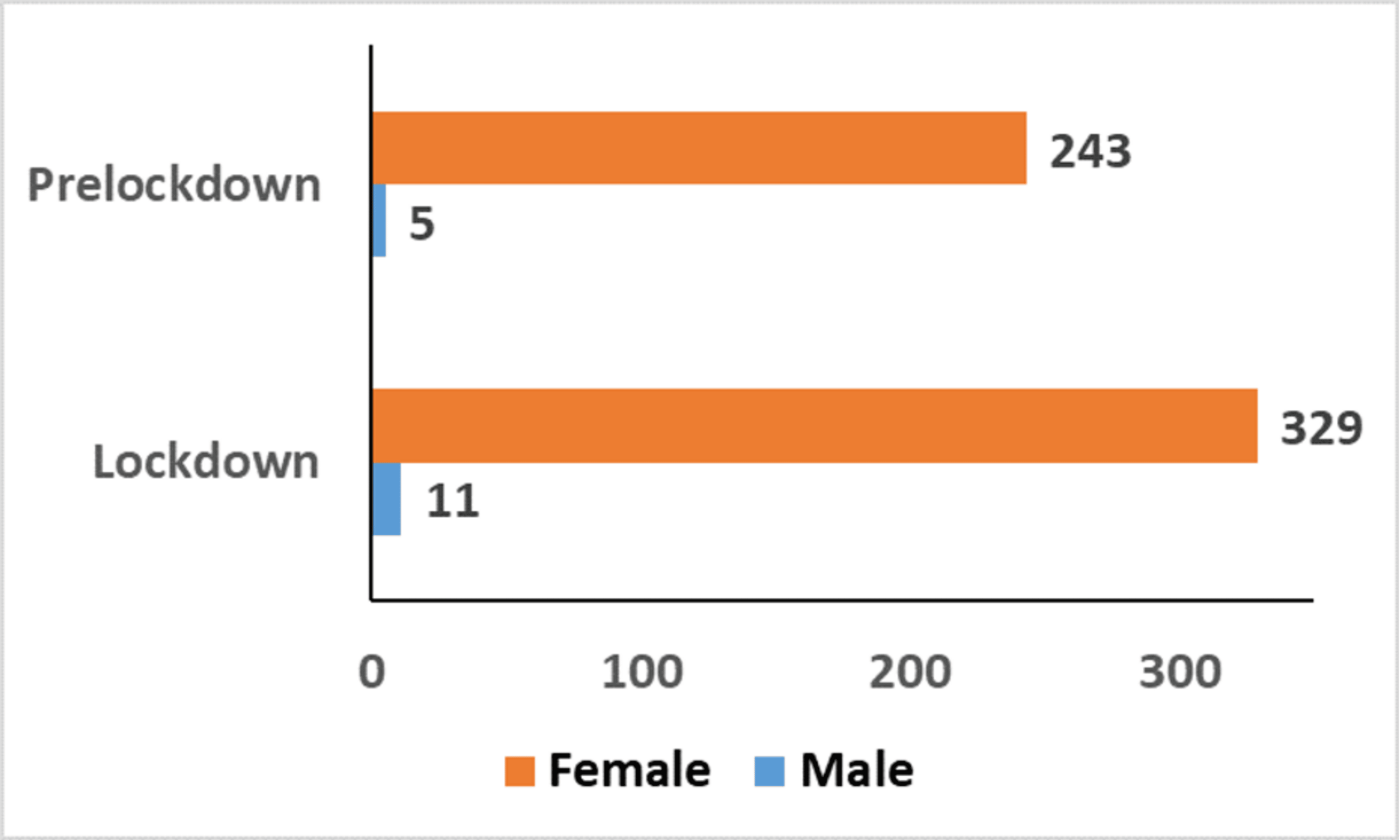
Sex Distribution

**Figure 2:**
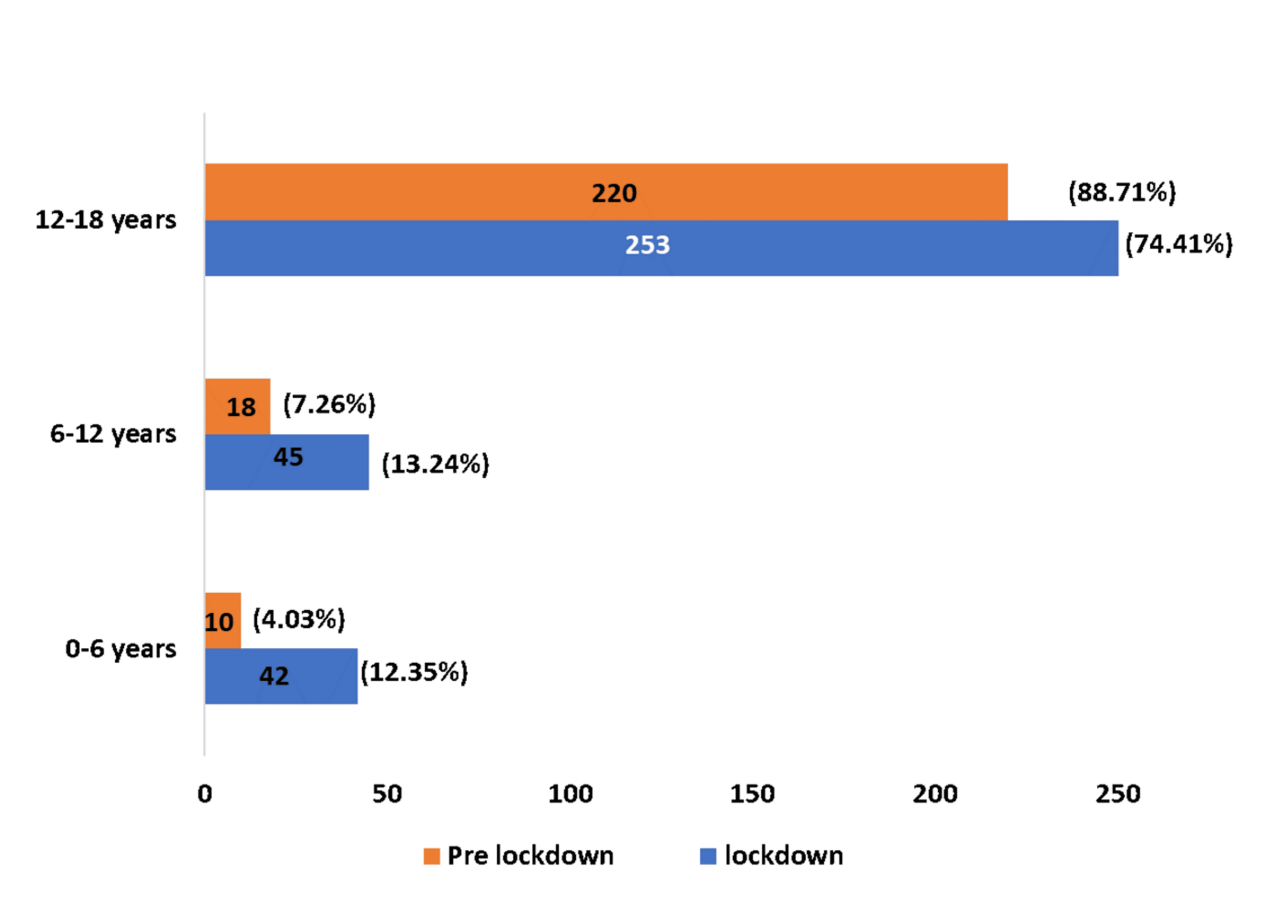
Age Distribution Chi-Square:19.6; P=0.00054<0.05. There is an association between age and the pre-lockdown and lockdown periods

The lockdown and the pre-lockdown periods saw a majority of female survivors of sexual abuse attending the Department of Forensic Medicine and Toxicology in the tertiary care center. A total of 248 survivors of child sexual abuse gave consent for the study during the pre-lockdown period, of which 243 were females and only five were males. In the lockdown period a total of 340 survivors gave consent for the study, of which 329 were females and 11 were males (Figure 1). The majority of the survivors, i.e., 88.71% during the pre-lockdown period and 74.41% during the lockdown period, were from the age group of 12 to 18 years (Figure 2). The chi-square test was positive, and an association was observed between the age groups and the period of the study (Figure 2).

In both the lockdown and the pre-lockdown periods, the majority of the survivors were pursuing their secondary education (Table 1). In the pre-lockdown period this percentage was 50.40%, and in the lockdown period it was 33.53% (Table 1). The lockdown period, however, presented an unfortunate picture of increased survivors among the students who were yet to pursue their formal education, along with the elementary school goers, the lower primary, and the upper primary students. Cliff's delta showed a small size effect (Table 1).

**Table 1:** Educational Status Cliff’s delta=-0.28, 72.2% overlap, small effect size.

	Pre-lockdown		Lockdown		Cliff’s Delta
Education Status	Frequency	Percent	Frequency	Percent	
Yet to begin	5	2.02	12	3.5	-0.28, 72.2% overlap, small effect size.
Elementary	5	2.02	30	8.8	
Lower primary	21	8.47	49	14.4	
Upper primary	57	22.98	95	27.9	
Secondary	125	50.40	114	33.5	
Illiterate	35	14.11	40	11.8	
Total	248	100.00	340	100.0	

**Figure 3:**
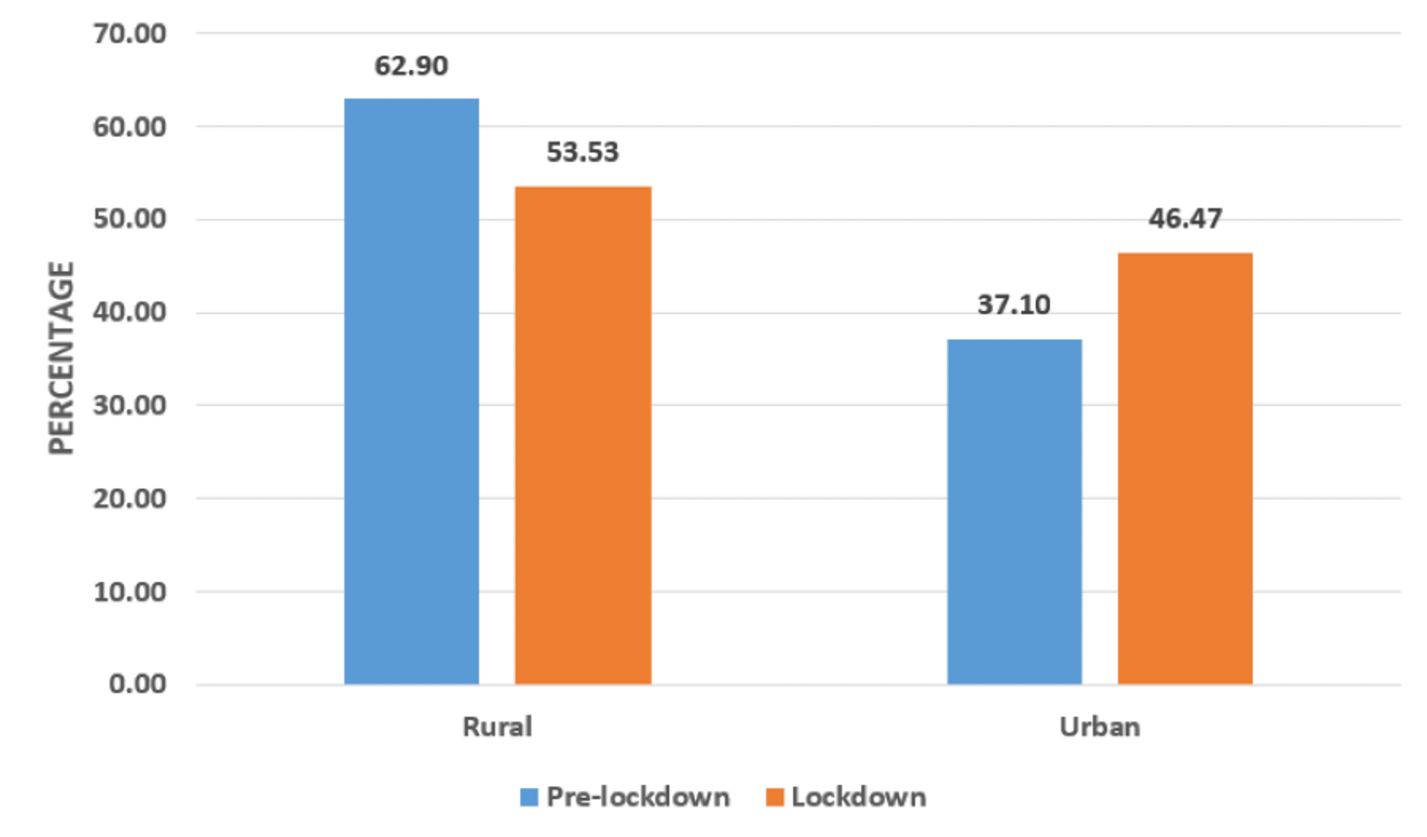
Place of Origin Chi-Square test is 19.6; P=0.023171<0.05. There is an association between pre-lockdown and lockdown periods

The pre-lockdown period marked a gross rural preponderance with 62.9% survivors (Figure 3). Urban-based survivors constituted only 37.1%. However, during the lockdown period, the predominance of the rural-based survivors was minimized. The rural-based survivors constituted 53.5% as compared to 46.5% of urban-based survivors in the lockdown period. The chi-square test was positive, and an association was observed between the place of origin and the periods of study (Figure 3).

**Figure 4:**
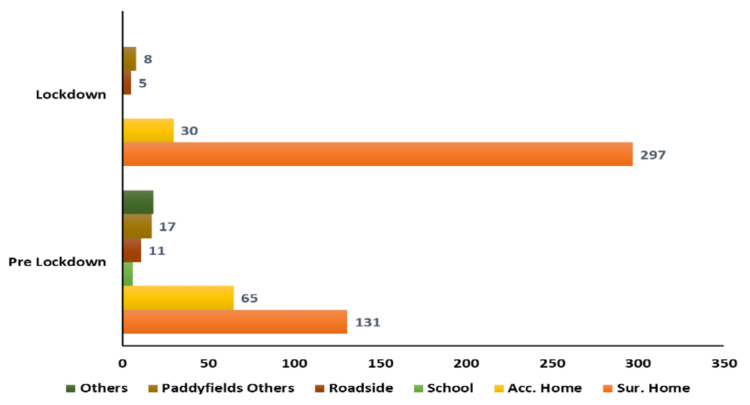
Place of Occurrence Cliff’s delta effect size is 0.39 (-0.39 to 0.84), corresponding to medium size effect

Survivor’s house as the place of occurrence of crime showed predominance in both the pre-lockdown (131) and the lockdown (297) periods (Figure 4). However, the lockdown period showed a higher margin of occurrence in the survivor’s home as compared to the pre-lockdown period. The accused’s house was the place that saw the second most occurrences of crime, after the survivor’s house, in both the pre-lockdown and the lockdown periods. Cliff’s delta showed a medium-sized effect (Figure 4).

**Figure 5:**
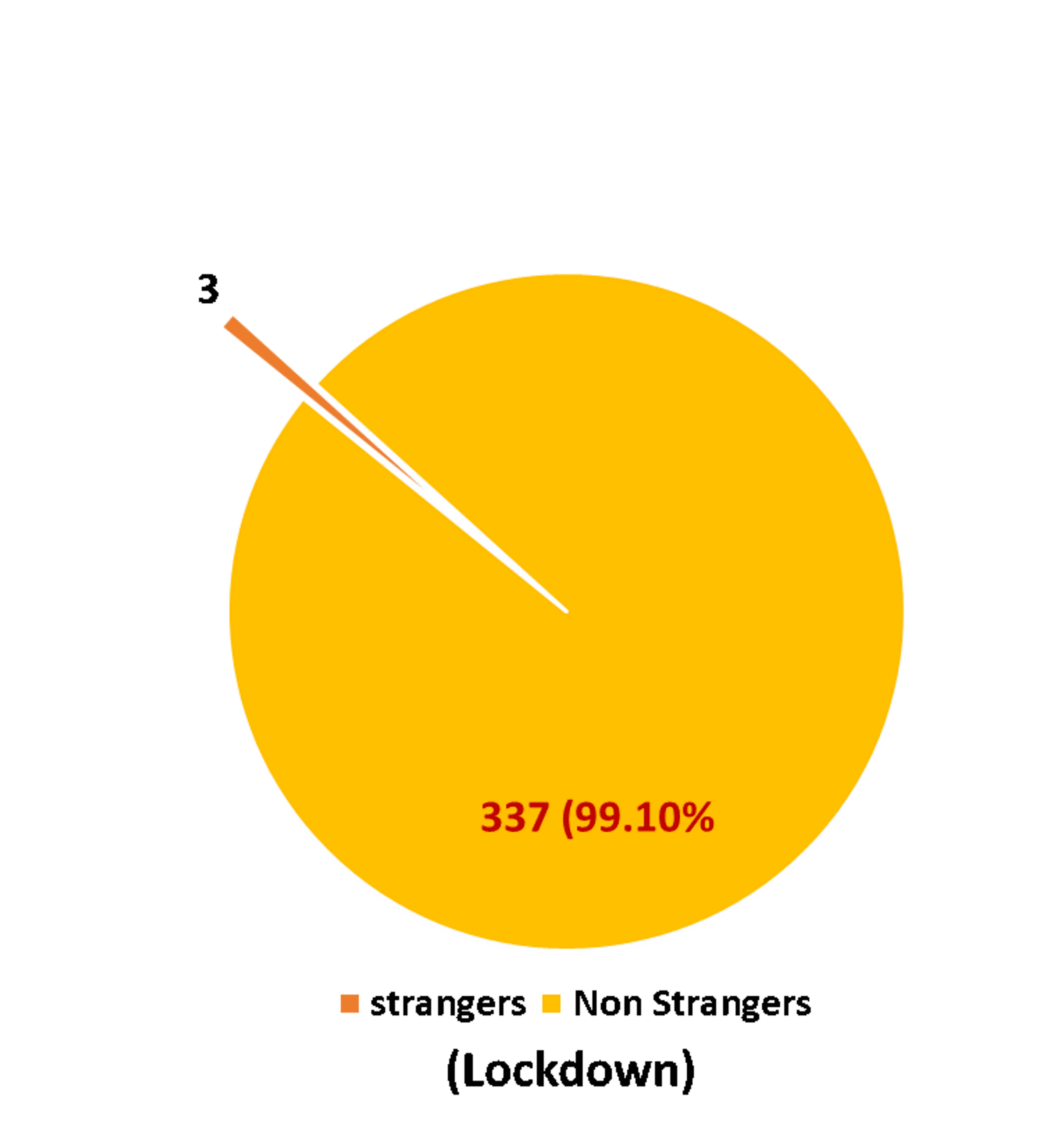
Strangers vs Non-strangers (Lockdown Period)

**Figure 6:**
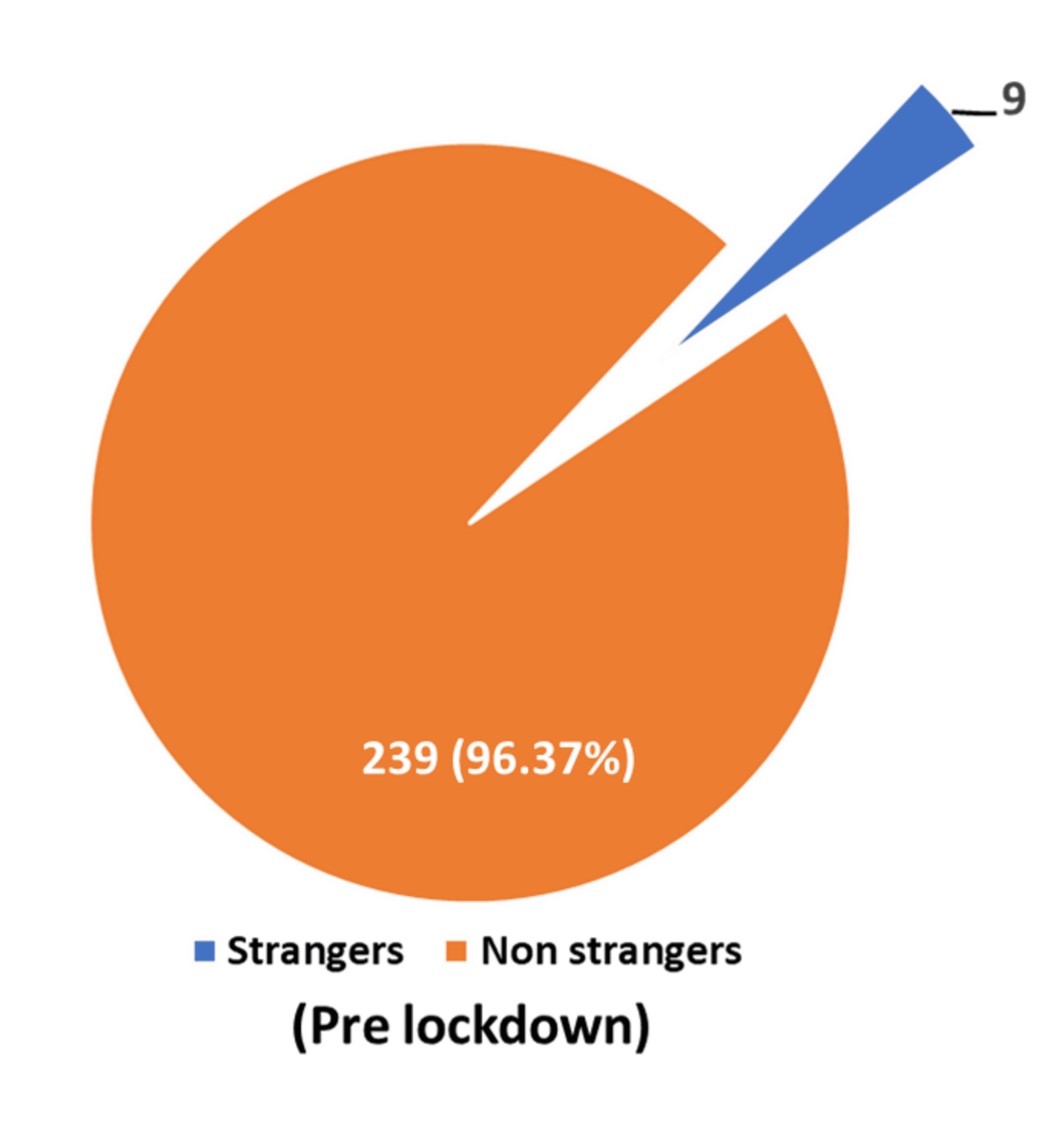
Strangers vs Non-strangers (Pre-lockdown Period)

Known faces as perpetrators of crime predominated in both the lockdown and pre-lockdown periods, with 99.1% and 96.37%, respectively (Figures 5, 6).

The nature of genital and other physical injuries of the survivors were the same in both the lockdown and the pre-lockdown period (Tables 2, 3). In the pre-lockdown period, maximum intact genitalia (anus and hymen) was seen in the 12-18 years age group and constituted 74.07% (Table 2). In the lockdown period, however, maximum intact genitalia (anus and hymen) was seen in the six to 12 year age group and was 37.35% (Table 3). Fresh tears were seen maximally in the 12-18 years of age group in both the pre-lockdown (80.0%) and lockdown (56.25%) periods (Tables 2, 3). Old tears were again seen in increased numbers in the 12-18 year age group in both the pre-lockdown (97.22%) and lockdown periods (96.17%) (Tables 2, 3). Other genital injuries, like those of the labia majora, labia minora, vagina, and posterior fourchette tears, did not show much variation in the pre-lockdown or lockdown periods.

**Table 2:** Genital Injury Comparison: Hymen and Anus (Pre-lockdown)

Age (years)	Intact	%	Fresh	%	Old	%
0-6 years	05	9.26	04	08.0	01	0.69%
6-12 years	09	16.66	06	12.0	03	2.09%
12-18 years	40	74.07	40	80.0	140	97.22%
Total	54	100.00	50	100	144	100

**Table 3:** Genital Injury Comparison: Hymen and Anus (Lockdown)

Age (years)	Intact	%	Fresh	%	Old	%
0-6 years	27.0	32.53	13.0	27.08	2.0	0.96
6-12 years	31.0	37.35	08.0	16.67	6.0	2.87
12-18 years	25.0	30.12	27.0	56.25	201.0	96.17
Total	83.0	100	48.0	100	209.0	100

**Figure 7:**
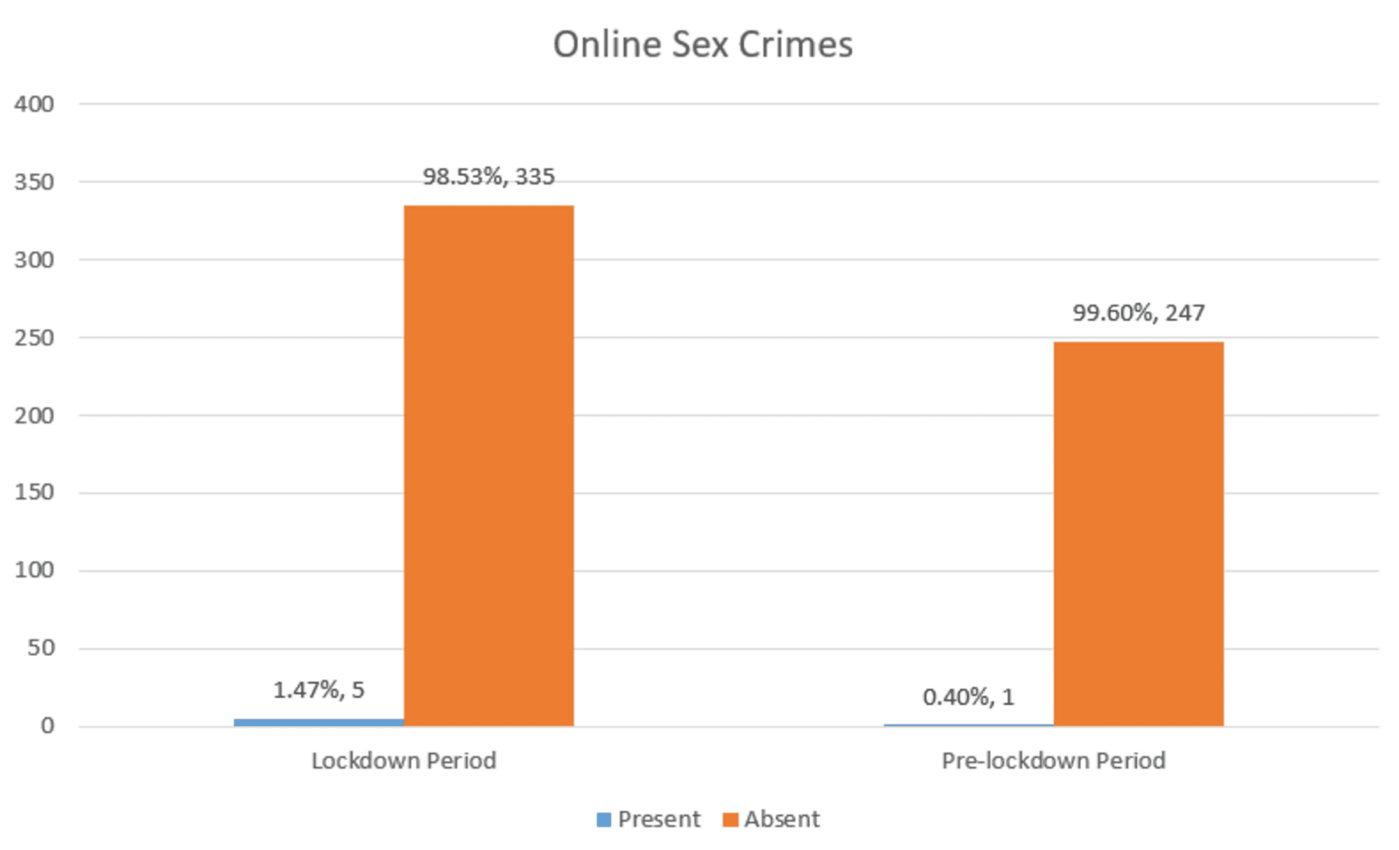
Online Sex Crimes

A mild increase of online sex crimes during the lockdown period of 1.4% as compared to 0.4% in the pre-lockdown period was observed (Figure 7).

Reporting of the cases was done maximally beyond seven days of the occurrence of crime in both the lockdown and the pre-lockdown periods (Table 4). However, 8.87% of crimes were reported in the first 24 hours during the pre-lockdown period as compared to only 1.76% in the lockdown period (Table 4). A similar trend followed for the 24- to 48-hour interval of occurrence of crime (Table 4).

**Table 4:** Time of Reporting Cliff's delta=-0.2 (-0.78 to 0.57) small size effect

Time of Reporting	Pre-lockdown	Lockdown	Cliff’s Delta
0-24 hours	22.00	6.00	-0.2 (-0.78 to 0.57) small size effect
%	8.87	1.76	
24-48 hours	41.00	27.00	
%	16.53	7.94	
48-72 hours	23.00	66.00	
%	9.27	19.41	
72 hours - seven days	18.00	32.00	
%	7.26	9.41	
More than seven days	144.00	209.00	
%	58.06	61.47	

## Discussion

Studies conducted across the world presented varied results regarding the rise or fall of the number of cases of child sexual abuse during the lockdown period as compared to the pre- or post-lockdown period. This study makes an inquisitive attempt to study the same in the social context of the state of Assam, India.

A rising trend of cases was observed in this study, with a 53.71% rise in cases as compared to the pre-lockdown period. Baron EJ et al., in a study conducted in the United States, found a 27% decrease in cases for the months of March and April 2020 [3]. School closure was found to be the main cause behind this [3]. As school personnel are considered to be the primary reporters of child maltreatment, their absence in the lives of the children led to lower rates of reporting [3]. In a study conducted by Augusti EM et al. in Norway, the prevalence of sexual abuse during COVID-19 was found to be 1.4%, with 1.6% of the survivors being girls and 1.2% being boys [4].

As per a study by Majeed-Ariss R et al. in the sexual assault referral center in Northwest England, monthly data for both children and adult survivors showed a decline during the lockdown period. It increased after the COVID-19 restrictions were lifted [5].

In our study, it was found that 74.41% of cases were from the age group of 12 to 18 years (Figure 2) during the lockdown period as compared to 88.71% in the pre-lockdown period (Figure 2). de Oliveira SM et al. found in their study conducted in Brazil that the age groups of two to nine years (38.1%) and 14 to 18 years (35.6%) had the maximum number of survivors in the lockdown period [6]. In a study conducted in Kenya by Ocheing W et al., it was found that there was a 73% to 122% increase in the number of cases of sexual violence in the age group of 10 to 17 years in the lockdown period as compared to non-pandemic times [7].

Our study showed a female predominance in both the pre-lockdown and lockdown periods (Figure 1). de Oliveira SM et al., in their study, stated that female survivors (76.7%) outnumbered male survivors [6]. Rockowitz S et al., in their study conducted in Kenya, found that both child and adult survivors were overwhelmingly female, 83% and 92%, respectively [8].

Child sexual abuse cases in the pre-lockdown period were mostly from the rural areas, as per our study (Figure 3). This predominance was hugely marginalized during the lockdown period, with 53.5% of the survivors only coming from a rural background (Figure 3). Ferraz AP et al., in their study, observed that most of the cases (84.05%) of sexual abuse occurred in the urban areas during COVID-19 [9].

The survivor’s house was the place of occurrence of crime in 87.35% of cases in this study during the lockdown period (Figure 4). Rockowitz S et al. found the perpetrator’s house to be the place of occurrence of crime in 41% of the cases involving children [8]. de Oliveira SM et al. found that sexual violence occurred mostly at the victim’s home (58.9%) [6]. The victim’s house dominated as the place of occurrence in the pandemic and non-pandemic times (27.99%) in a study conducted by Ferraz AP et al. [9]. Owusu-Addo E et al. reported that 58.8% of the incidents took place in another person’s house and 17.6% in the victim’s house [10].

Known faces dominated as perpetrators of crime in the present study during the lockdown and pre-lockdown periods (Figures 5, 6). Owusu-Addo E et al. reported that 31.30% were acquaintances, 25% romantic partners, 18.8% neighbors, 12.5% peers, 6.3% family members, and 6.1% others [10]. Rockowitz S et al. found 76% of the children were abused by people known to them in the lockdown period [8]. Ferraz AP et al. found that known faces, as perpetrators, occupied 51.20% during the lockdown period [9].

A study by Owusu-Addo E et al. in Ghana among the adolescent groups found that 52.4% had completed junior high school, 29.1% vocational education, 12.1% primary, and 0.4% tertiary; 63.3% of the survivors reported being in school [10]. Contrary to this study, the majority of the survivors were attending their secondary-level education at the time of the occurrence of the crime during the lockdown period and also in the pre-lockdown period in our study (Table 1). However, the disturbing fact in our study remains that more children belonged to the category of ‘yet to begin’ and those pursuing elementary education as compared to the pre-lockdown period (Table 1).

Children were restricted to their homes during the lockdown period and were forced to attend online classes, resulting in increased exposure to the internet (Figure 7). This rendered the children vulnerable to online crimes, sex-linked or otherwise. A slight escalation in online sexual crime was found during the lockdown period as compared to the pre-lockdown period in our study (Figure 7). In a study conducted in England, Harris M et al. found a 17% increase in online sexual crimes during the lockdown period as compared to the pre-lockdown period [11]. Santos CC et al., in their study, found a 108% increase in child pornography in the state of Pernambuco, Brazil, shortly after the pandemic declaration [12]. A report shared by Interpol highlighted the rampant use of child pornography during the lockdown period [13]. As per the report, sex offenders with technical expertise to administrate forums have had more time to create new ones [13]. The users, on the other hand, benefited from the availability of more materials on the net [13]. Further, the report claimed that children whose parents were hospitalized or expired during the lockdown period and were placed under the care of others suffered a lot [13]. Live streaming of pornography with the child being locked with facilitators and increased economic hardships further worsened the matter [13].

Maximal reporting of the cases was done beyond seven days of the occurrence of crime during both the lockdown and pre-lockdown periods in our study (Table 4). However, compared to the lockdown period, there was comparatively more early reporting done within the first 24 to 48 hours in the pre-lockdown period (Table 4). Paramasivan K et al., in a study conducted in Tamil Nadu, India, reported that the median delay in reporting during the pre-pandemic area was six days [14]. This increased to 40 days and 14 days in the mild intervention period and post-intervention phases of the second wave, respectively [14]. Delay in reporting was a notable phenomenon in the lockdown period as compared to the pre-lockdown period in our study (Table 4).

The limitation of the study is precipitated by its single-center design, hindering generalization to the broader population of northeast India. Future studies of multi-centric origin including the post-pandemic period are recommended.

## Conclusions

Most child sexual abuse is opportunistic rather than carefully planned, and abuse tends to follow a path of least resistance. The data on sexual abuse revealed in this study are alarming and call for strict intervention. This is because of the fact that children are unsafe in their own homes. The fear of these survivors becoming potential perpetrators of tomorrow cannot be ruled out. The risk of relational violence among them is a common finding in many studies. The lockdown period due to COVID-19 is a difficult phase in the history of mankind where social and economic restrictions disrupted the whole social milieu. The smaller number of male survivors in most of the studies is not an affirmative indication but a reflection of the attitude of the society towards the male survivors. To tide over this distressful situation, emphasis should be laid on better circulation of information and services required to protect children against sexual abuse. Social and community workers should be asked to be on vigil during such times. Child, juvenile, and family courts should remain functional with proper protective measures during such times. The education policy should educate children on warning signs of sexual abuse. The overburdened health professionals, during such pandemics, may miss signs of sexual violence due to increased workload. This serious observation needs to be given due consideration in times of disaster. The Child Helpline and other social support systems need to be activated in such times of disaster.
